# The role of fibroblast-neutrophil crosstalk in the pathogenesis of inflammatory diseases: a multi-tissue perspective

**DOI:** 10.3389/fimmu.2025.1588667

**Published:** 2025-05-23

**Authors:** Chen Cai, Lanxi Guan, Chenhao Wang, Runjie Hu, Lingling Ou, Qianzhou Jiang

**Affiliations:** ^1^ Department of Endodontics, School and Hospital of Stomatology, Guangdong Engineering Research Center of Oral Restoration and Reconstruction & Guangzhou Key Laboratory of Basic and Applied Research of Oral Regenerative Medicine, Guangzhou Medical University, Guangzhou, China; ^2^ Department of Periodontics, School and Hospital of Stomatology, Guangdong Engineering Research Center of Oral Restoration and Reconstruction & Guangzhou Key Laboratory of Basic and Applied Research of Oral Regenerative Medicine, Guangzhou Medical University, Guangzhou, China

**Keywords:** neutrophils, fibroblasts, inflammation, NETs, chemokines, cytokines

## Abstract

Neutrophil-fibroblast crosstalk drives inflammatory pathology across organ systems through both shared and tissue-specific mechanisms. This review synthesizes evidence from skin, lung, gut, cardiovascular, joint, sinus, and oral diseases, revealing conserved molecular pathways where fibroblasts secrete chemokines (CXCL1/8/12) to recruit neutrophils, which, in turn, release neutrophil extracellular traps (NETs), elastase, and cytokines to modulate fibroblast function. Additionally, we identify critical tissue-specific differences, including the predominance of IL-36 signaling in COPD, IL-17-carrying NETs in systemic lupus erythematosus (SLE) and pulmonary fibrosis, and specialized fibroblast subpopulations, such as IDO1+ cells in CRSwNP and TNFRSF21+ cells in periodontitis. Translational insights highlight the therapeutic potential of targeting IL-17, NETs, and fibroblast subpopulations, though tissue-specific risks necessitate precision strategies. Future therapeutic efforts should focus on developing precision-targeted interventions that address organ-specific mechanisms to overcome treatment resistance in inflammatory disorders.

## 1 Introduction

Neutrophils and fibroblasts are two critical cell types involved in inflammatory responses and tissue remodeling across various organ systems. In recent years, growing evidence has highlighted the complex bidirectional interactions between these cells, revealing their pivotal roles in the pathogenesis of inflammatory diseases. Neutrophils, as the first line of defense in innate immunity, not only eliminate pathogens but also orchestrate inflammatory responses by releasing cytokines, chemokines, and NETs (1). Fibroblasts, the primary mesenchymal cells in connective tissues, are responsible for maintaining tissue homeostasis and regulating the composition of the extracellular matrix (ECM) (2). While their individual roles in inflammatory conditions are increasingly recognized, a significant knowledge gap remains regarding how neutrophil-fibroblast crosstalk varies across different tissues and disease states. Current clinical interventions targeting broad-spectrum molecules like IL-17 can yield unpredictable outcomes, with treatments effective in one tissue potentially exacerbating inflammation in others. This inconsistency suggests that tissue-specific mechanisms underlying neutrophil-fibroblast interactions critically influence disease pathogenesis and treatment responses. Understanding how fibroblast heterogeneity, neutrophil functional plasticity, and systemic factors modulate these interactions is vital for developing targeted therapies. This review integrates multi-organ evidence to map both universal and tissue-specific crosstalk mechanisms and discusses potential translational challenges, contributing to the evolving field of precision immunology and informing future clinical research.

## 2 Skin

The skin is the largest organ of the human body and serves as an important barrier defending against the invasion of external pathogens (3). Fibroblasts regulate the recruitment and activation of neutrophils in skin diseases through multiple mechanisms, with IL-17 and IL-1 acting upstream in this process. CXCL12+ fibroblasts, located in the reticular dermis, express adipocyte lineage markers and can sense IL-17 and TNF-α stimulation, releasing CXCL12 in an NFKBIZ-dependent manner to promote neutrophil recruitment (4). The CXCL12+ fibroblast subset in human psoriatic lesions highly expresses neutrophil chemokines, which are significantly reduced after targeted IL-17 therapy (4). In mouse models, specific deletion of the Il17ra gene in fibroblasts significantly reduced neutrophil recruitment and exacerbated *S. aureus* infection, indicating the importance of fibroblast recognition of IL-17 signals for neutrophil recruitment and infection clearance in skin lesions (4). Another study using single-cell RNA sequencing found that fibroblasts are the main non-immune cells accumulated at the site of C.albicans skin infection, with high expression of the IL-17ra gene and chemokines involved in neutrophil recruitment, such as CXCL1, CXCL2, CXCL5, CXCL12, Lcn2, and Il33 (5). In addition, Cordier-Dirikoc et al. (6) found that dermal fibroblasts are highly sensitive to IL-1 released by keratinocytes after skin injury and can express high levels of IL-8 to recruit neutrophils after IL-1 stimulation. The ability of IL-1-stimulated fibroblasts to recruit neutrophils has been verified in *in vitro* cell migration experiments and IL-1R1-deficient mouse models of epidermal injury, with IL-1R1-deficient mice showing reduced expression of inflammatory mediators and neutrophil infiltration in the skin (6).

The phenotype and function of fibroblasts exhibit heterogeneity in different skin diseases. Li et al. (7) identified CXCL1+ fibroblasts across multiple skin conditions (healthy, psoriasis, atopic dermatitis, lupus, scleroderma, and scars) that function in neutrophil recruitment. They further demonstrated that psoriasis-specific COL6A5+ fibroblasts differentiate into CXCL1+ fibroblasts, subsequently promoting neutrophil chemotaxis and tissue infiltration. There is a distinct subset of CXCL1+ fibroblasts in SLE lesions that not only recruit and activate neutrophils, increase the production of inflammatory mediators, reactive oxygen species, and NETs, but also promote neutrophil transformation to a low-density phenotype and delay apoptosis. Serum amyloid A1 secreted by CXCL1+ fibroblasts is a key activating factor for neutrophils, while activated neutrophils further induce the differentiation of CXCL1+ fibroblasts by secreting IL-1β to activate the NF-κB pathway, forming a positive feedback loop (8).

NETs can affect the functions of various cells, including macrophages, lymphocytes, dendritic cells, and fibroblasts (9), and their regulatory effects on fibroblasts are bidirectional. Low concentrations of NETs can activate the ILK/PI3K/AKT signaling pathway of fibroblasts by binding to CCDC25, promoting cell proliferation and migration (10). However, over-activation of NETs interferes with healing factor regulation in fibroblasts by enhancing the NF-κB inflammatory response and suppressing the Wnt/β-catenin pathway, thereby attenuating the fibrosis process essential for wound healing (11). NETs containing IL-17 have been found to lead to the activation and differentiation of human fibroblasts, inducing collagen production. The presence of such IL-17A-carrying NETs in skin biopsies from SLE patients suggests that they may contribute to fibrosis in SLE by activating fibroblasts (12). In addition, human neutrophil peptides (HNPs), produced by neutrophils and monocytes as members of the α-defensin family, have strong antimicrobial activity. Studies have shown that HNP1–3 can induce the proliferation and activation of human dermal fibroblasts without producing cytotoxicity, with HNP1 showing a stronger effect on promoting cell proliferation and type I collagen production compared to other HNPs (13).

## 3 Lung

Fibroblasts and neutrophils are believed to play critical roles in the process of pulmonary fibrosis. Fibroblasts are the primary source of ECM and are considered key contributors to fibrotic lung diseases (14). Additionally, fibroblasts can respond to various pathogenic stimuli such as bacterial or viral infections and toxic gases. They produce typical neutrophil-recruiting cytokines like IL-6 and IL-8, thereby participating in the excessive accumulation of neutrophils in asthma and chronic obstructive pulmonary disease (COPD) (15, 16). At the same time, neutrophils also influence the activation and differentiation of fibroblasts through multiple pathways.

As members of the IL-1 superfamily, IL-36 cytokines can act on lung fibroblasts during the COPD process, stimulating them to release large amounts of chemokines, pro-inflammatory cytokines, and proteases that promote disease progression. Koss et al. (17) used a mouse COPD model to reveal that neutrophils are the main source of IL-36γ, which can activate alveolar and interstitial fibroblasts to produce IL-1, CXCL1, GM-CSF, MMP9, and IL-36 to promote inflammatory responses and fibrosis. Another study confirmed that IL-36γ stimulation of small airway fibroblasts (SAF) promotes release of CXCL8, IL-6, CXCL1, GM-CSF, MMP2, and MMP9, leading to neutrophil recruitment and enhanced inflammatory responses (18). In *in vitro* models, IL-36 receptor antagonist (IL-36Ra) or therapeutic antibodies inhibiting IL-36R-mediated signaling disrupted communication between small airway epithelial cells and SAF, reducing CXCL1 release. This suggests that targeting IL-36 signaling may represent a potential strategy for treating COPD (18).

Circadian rhythm is another factor influencing the interaction between fibroblasts and neutrophils in pulmonary diseases. In lung tissues of mice stimulated with LPS at dusk, the expression of inflammatory factor IL-1β and chemokine CXCL5 was upregulated, accompanied by increased neutrophil recruitment (19). Upon IL-1β stimulation, CXCL5 expression was markedly enhanced in both primary and immortalized lung fibroblasts lacking circadian clock protein BMAL1. Subsequently, conditioned medium derived from IL-1β-treated Bmal1-/- fibroblasts exhibited enhanced chemotactic activity, promoting greater neutrophil migration compared to Bmal1+/+ controls. Mechanistically, elevated phosphorylation of NF-κB p65 was observed in BMAL1-deficient cells, with NF-κB inhibition effectively attenuating both CXCL5 production and neutrophil recruitment (19).

NETs and their components play a role in promoting the activation of lung fibroblasts and their differentiation into myofibroblasts, which are the main phenotype that triggers pulmonary fibrosis (20). Activated lung fibroblasts or differentiated lung myofibroblasts can resist apoptosis and alter the quantity and composition of the extracellular matrix, contributing to the progression of pulmonary diseases (21). Failure to clear NETs, coupled with extracellular matrix accumulation and the production of pro-fibrotic cytokines, impedes wound healing, resulting in the loss of lung structure and function (14). A study found that lung fibroblasts treated with NETs showed increased expression of connective tissue growth factor, collagen production, and enhanced proliferation/migration, which could be blocked by NETs inhibitors (22). Further research confirmed that IL-17 was expressed in NETs and promoted the fibrotic activity of differentiated lung fibroblasts, but did not promote their differentiation. IL-17 had no direct effect on the activation and differentiation of lung fibroblasts, indicating that DNA and histone-induced responses were crucial for IL-17-driven fibrosis (22). In addition, neutrophil elastase (NE), matrix metalloproteinases (MMPs), and MMP tissue inhibitors (TIMPs) released by NETs can regulate extracellular matrix components. Among them, NE can enter fibroblasts, stimulate fibroblast growth, affect their contractile properties, and promote their differentiation into myofibroblasts. This process involves the TLR9-miR-7-Smad2 signaling pathway (23, 24).

## 4 Gastrointestinal tract

The pathogenesis of inflammatory bowel disease (IBD), particularly Crohn’s disease (CD), involves abnormal interactions between the intestinal immune system and mesenchymal cells. In an inflammatory microenvironment rich in NETs, neutrophils from CD patients exhibit pro-fibrotic properties. These neutrophils are capable of activating intestinal fibroblasts and promoting collagen release, a process mediated by the IFN-α signaling pathway. Moreover, activated fibroblasts secrete IL-8, which attracts neutrophil infiltration into the intestinal mucosa, thereby maintaining this fibrotic loop (25).

IL-17 expression is enhanced in the intestinal tissues of CD patients, which can induce intestinal fibroblasts to express the transcription factor NFKBIZ and pro-inflammatory chemokine CXCL1, thereby affecting fibroblast activity and neutrophil chemotaxis (26). Dragoni et al. (27) discovered that NETs and activated fibroblasts exhibit spatial overlap in the ulcerated regions of the CD ileum. Fibroblasts treated with NETs upregulated pro-fibrotic gene expression and activated TLR signaling cascades, particularly the TLR2/NF-κB pathway, resulting in enhanced fibroblast proliferation and collagen production.

Through RNA sequencing of the inflammatory response in IBD tissues, Friedrich et al. (28) found that activated fibroblasts at the ulcer base exhibit properties of recruiting neutrophils, a process dependent on IL-1 receptor rather than TNF. In patients who are non-responsive to multiple treatments, the expression of pathology-associated neutrophil and fibroblast signature genes is elevated. This suggests that targeting neutrophil-fibroblast interactions may be a potential strategy to overcome treatment resistance. Further research has revealed an increased presence of NRG1+IL1R1+ fibroblasts in the intestinal tissues of IBD patients. Following inflammation-induced epithelial damage, neutrophil-secreted IL-1β can inhibit intestinal epithelial repair. This occurs through interference with the secretion of reparative neuregulin-1 by IL1R+ fibroblasts, ultimately exacerbating intestinal inflammation (29).

## 5 Cardiovascular

Vascular diseases develop through complex interactions between various cell types within the vessel wall. Research has shown that the walls of cerebral arteries consist of vascular smooth muscle cells (vSMCs) and perivascular fibroblasts, which not only provide structural support but also regulate the behavior of endothelial cells during angiogenesis and the maintenance of vascular homeostasis (30). In ischemic conditions, increased NET levels activate fibroblasts, which subsequently promote smooth muscle cell proliferation through the Wnt5a pathway. Inhibition of Wnt5a has been shown to alleviate vascular remodeling and subsequent tissue damage in mouse models of ischemia (31).

IFN-β has been found to exert a bidirectional regulatory effect on myocardial inflammation. Bolívar et al. (32) demonstrated that IFN-β activates STAT1 in cardiac fibroblasts, leading to enhanced expression of the chemokines MCP-1 and IP-10, thereby increasing neutrophil migration. However, IFN-β can also activate STAT3, inducing the secretion of the anti-inflammatory cytokine IL-10 (32). Another study revealed that co-culturing neutrophils and cardiac fibroblasts *in vitro* enhanced the production of certain extracellular matrix proteins while specifically reducing collagen synthesis (33). Furthermore, neutrophils are necessary for inducing a transient upregulation of TGF-β1 expression in fibroblasts, which is crucial for terminating the pro-inflammatory phase and allowing the formation of a mature scar during the repair process (33). These findings suggest that neutrophils play a dual role in the healing process following myocardial infarction, both initiating and terminating the inflammatory events that control and regulate tissue repair.

In chronic thromboembolic pulmonary hypertension (CTEPH), neutrophil inflammation contributes to fibroblast activation by enhancing TGF-β signaling and through the release of NETs. This leads to fibrotic remodeling of thrombi. Targeting the DNA component of thrombus NETs may represent a novel strategy for treating thrombosis and preventing its sequelae (34).

## 6 Joint

The pathogenesis of joint diseases such as rheumatoid arthritis (RA) involves complex interactions between various cellular components in the synovial microenvironment. Studies have shown that the synovial fluid of RA patients is rich in NETs, which contain citrullinated peptides that can be internalized by fibroblast-like synoviocytes (FLS) through the RAGE-TLR9 pathway (35). This promotes the transformation of FLS into an inflammatory phenotype and upregulates the expression of MHC class II molecules. The internalized arthritogenic NETs peptides are loaded onto the MHC class II molecules of FLS and presented to antigen-specific T cells (35). This finding highlights the crucial role of FLS in the pathogenesis of RA, as they uptake and present NETs-citrullinated peptides, activating autoimmune responses and leading to tissue damage.

Research by Wu et al. (36) has also confirmed that IgG containing anti-citrullinated protein antibodies (ACPA) can stimulate neutrophils to produce NETs, activating FLS to upregulate the expression of proinflammatory factors such as IL-6 and IL-8. IL-33, another proinflammatory cytokine, can activate neutrophils to produce NETs, and the reactivity of neutrophils to IL-33 stimulation is enhanced in RA patients. Evidence from both *in vivo* and *in vitro* studies demonstrates that NETs induce IL-33 and CXCL8 secretion by FLS via TLR9 activation. This establishes an IL-33-mediated positive feedback loop in the RA synovium, characterized by enhanced neutrophil recruitment and subsequent NETs formation (37). At the molecular level, NETs can induce pyroptosis and phenotypic transformation of RA-FLS through the NF-κB/caspase-3/GSDME pathway. Knocking down GSDME significantly alleviates NET-induced pyroptosis and pathogenic behavior of RA-FLS (38). Recent findings have revealed that NETs activate ATP-citrate lyase (ACLY) in RA-FLS, driving lipogenesis, cell proliferation, migration, invasiveness, and NF-κB-mediated inflammation, ultimately exacerbating synovial inflammation and joint damage in RA (39).

Further research has discovered that FLS derived from RA patients can induce neutrophil migration by secreting soluble mediators and prolong their survival in a cell contact-dependent manner. However, the impact of FLS on neutrophil phenotypes and functions, such as reactive oxygen species (ROS) production and degranulation, is limited (40).

## 7 Sinus

Recent research has unveiled the critical roles of fibroblasts and neutrophils in the development of chronic rhinosinusitis with nasal polyps (CRSwNP), involving the IL-1 signaling pathway. Using single-cell sequencing technology, researchers have identified a specific IDO1+ fibroblast subpopulation induced by IL-1 signaling, promoting neutrophil recruitment by releasing CXCL1/2/3/5/6/8. IL-1β, a major proinflammatory cytokine involved in IL-1 signaling, can induce the differentiation of primary fibroblasts into IDO1+ fibroblasts (41). In a lipopolysaccharide-induced neutrophilic CRSwNP mouse model, blocking IL-1β activity with an IL-1β receptor antagonist significantly reduced the number of IDO1+ fibroblasts and alleviated nasal inflammation (41). This finding reveals the function of the IDO1+ fibroblast subpopulation in neutrophil recruitment and provides a basis for IL-1-targeted intervention in neutrophilic inflammation in CRSwNP.

Further research has discovered that granzyme K+ (GZMK+) CD8+ T cells are significantly increased in CRSwNP tissues, with a phenotype distinct from that of cytotoxic GZMB+ effector CD8+ T cells. GZMK+ CD8+ T cells express CXCR4 and can interact with CXCL12-secreting fibroblasts, inducing the latter to produce neutrophil chemokines through GZMK. This GZMK+ CD8+ T cell-fibroblast crosstalk has also been found in other inflammatory diseases (42). The study elucidates a novel mechanism by which the GZMK+ CD8+ T cell-fibroblast axis mediates neutrophil infiltration in CRSwNP. Zhang et al. (43) have also revealed that CXCL8+ neutrophils in CRSwNP tissues can secrete the mediator oncostatin M (OSM) to mediate communication with other cells. OSM stimulates fibroblasts to enhance IL-13-mediated production of CCL26 and periostin, revealing the mechanism by which neutrophils amplify type 2 inflammatory responses and participate in the pathogenesis of CRSwNP.

## 8 Oral cavity

The development of oral diseases, especially periodontitis, is a complex pathological process involving interactions between various immune cells and mesenchymal cells. Fibroblasts, particularly gingival fibroblasts, can secrete chemokinessuch as CXCL1, CXCL2, CXCL12, CXCL16 when stimulated by inflammatory factors like IL-1β and IL-36γ in an inflammatory state. These chemokines act on chemokine receptors such as CXCR2/4 on the surface of neutrophils, recruiting them to infiltrate the inflamed tissue (44–47).

Caetano et al. (48) employed single-cell RNA sequencing, spatial transcriptomics, and immunofluorescence *in situ* hybridization to identify a distinct pro-inflammatory stromal signature in the junctional epithelium of periodontal tissue. This signature was characterized by the specific expression of genes involved in neutrophil activation and degranulation. They identified a specific subset of fibroblasts that highly express key genes such as CXCL8, CXCL10, and ALOX5AP, which promote neutrophil infiltration and may also participate in pathological angiogenesis. Chen et al. (49) found a significantly expanded subset of TNFRSF21+ fibroblasts in periodontitis, which highly express the neutrophil chemokines CXCL1, CXCL2, CXCL5, and CXCL6.

Neutrophil infiltration can cause tissue damage by releasing NETs, and this process can be regulated by fibroblasts. Compared to normal gingival tissue, fibroblasts isolated from inflamed gingival tissue can induce more NETs formation *in vitro* and promote the polarization of macrophages into the CD86+ M1 inflammatory phenotype (50, 51). Qiu et al. (50) employed single-cell sequencing and machine learning to identify a neutrophil subset (NrNeu) associated with NETs formation. Their findings revealed that gingival fibroblasts exhibited the highest level of cellular interactions with NrNeu. Furthermore, they discovered that fibroblasts regulate NETs formation during periodontitis through the macrophage migration inhibitory factor (MIF)-CD74/CXCR4 axis. Additionally, infiltrating neutrophils cooperate with fibroblasts to activate type 3 innate lymphoid cells in periodontal tissue, resulting in enhanced IL-17 production and subsequent exacerbation of inflammatory responses and bone resorption (52).

Studies have also found significantly elevated levels of IL-36γ in periodontitis, which can specifically activate gingival fibroblasts, leading to neutrophil chemotaxis. Inhibiting IL-36γ signaling can reduce neutrophil infiltration and bone resorption, revealing another axis of fibroblast-neutrophil interaction in periodontitis (44). Zhang et al. (53) discovered that salicylic acid can activate the G protein-coupled receptor Tas2r143 in mouse fibroblasts, thereby inhibiting LPS-induced expression of chemokines CXCL1, CXCL2, and CXCL5. Salicylic acid can also suppress periodontal bone loss, inflammation, and neutrophil infiltration in mice with periodontitis, providing a potential strategy for targeting fibroblasts in the treatment of periodontitis.

In addition to direct cellular interactions, systemic factors also influence the fibroblast-neutrophil axis and, consequently, the progression of periodontitis. Wang et al. (54) used leptin receptor-deficient mice (db/db) to find that fibroblasts expand during the process of periodontitis in type 2 diabetic mice, with significant upregulation of the IL-17 receptors Il17ra and Il17rc, the IL-1β receptor IL1R1, and the neutrophil chemokines CXCL1, CXCL5, CXCL10, and CXCL12. Another study found that in a diabetic state, the regulatory function of periodontitis fibroblasts on immune cells is enhanced, especially their interaction with neutrophils through the CXCL12-CXCR4 axis (55). Shinjo et al. (56) discovered that after bacterial LPS stimulation, insulin can enhance the secretion of CXCL1 by mouse and human gingival fibroblasts to recruit neutrophils for bacterial clearance through the Akt pathway and NF-κB activation. In diabetic mice with insulin resistance and insulin receptor signaling deficiency, this process is weakened, leading to delayed neutrophil recruitment and aggravated alveolar bone destruction during periodontitis.

Mo et al. (57) used single-cell sequencing to reveal differences in cellular composition and molecular environment between peri-implantitis and periodontitis. They discovered the involvement of inflammation-promoting fibroblasts and a predominance of CXCL8+ fibroblast-CXCR2+ neutrophil interactions in peri-implantitis compared to periodontitis, underlining the enhanced host response in peri-implantitis.

## 9 Clinical translation: applications and challenges

**Figure 1:**
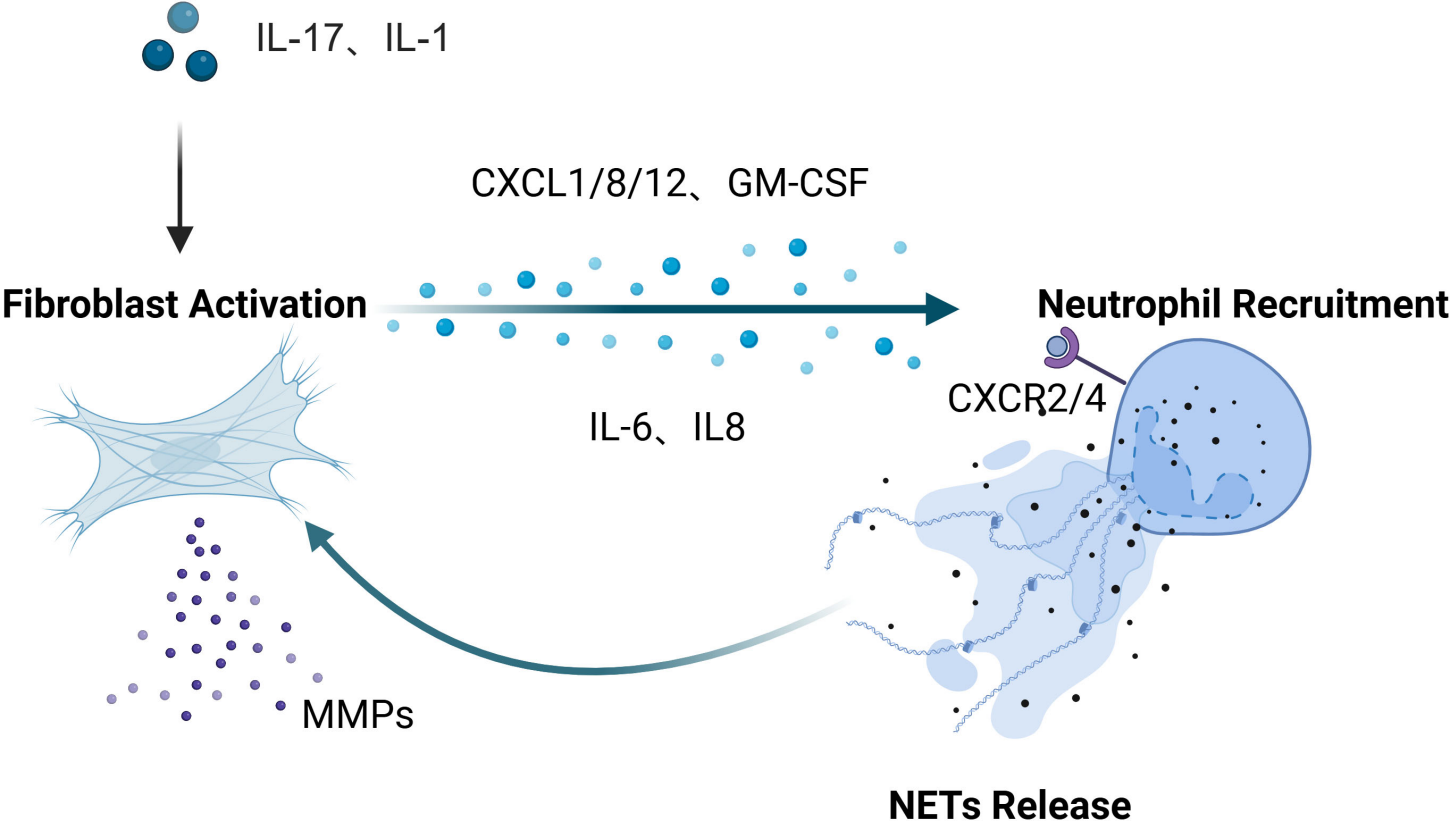
A conserved inflammatory axis is evident throughout all tissues: under stimulation by upstream inflammatory mediators such as IL-17 and IL-1β, fibroblasts secrete chemokines including CXCL1, CXCL2, CXCL8, and CXCL12, which bind to neutrophil surface receptors (primarily CXCR2 and CXCR4) to orchestrate neutrophil recruitment and infiltration into inflammatory sites. Reciprocally, neutrophils modulate fibroblast phenotype and function through the release of NETs, elastase, and pro-inflammatory cytokines, establishing a positive feedback loop that perpetuates and amplifies inflammatory responses. Created in BioRender. cai, c. (2025) https://BioRender.com/6hm5abd.

**Figure 2:**
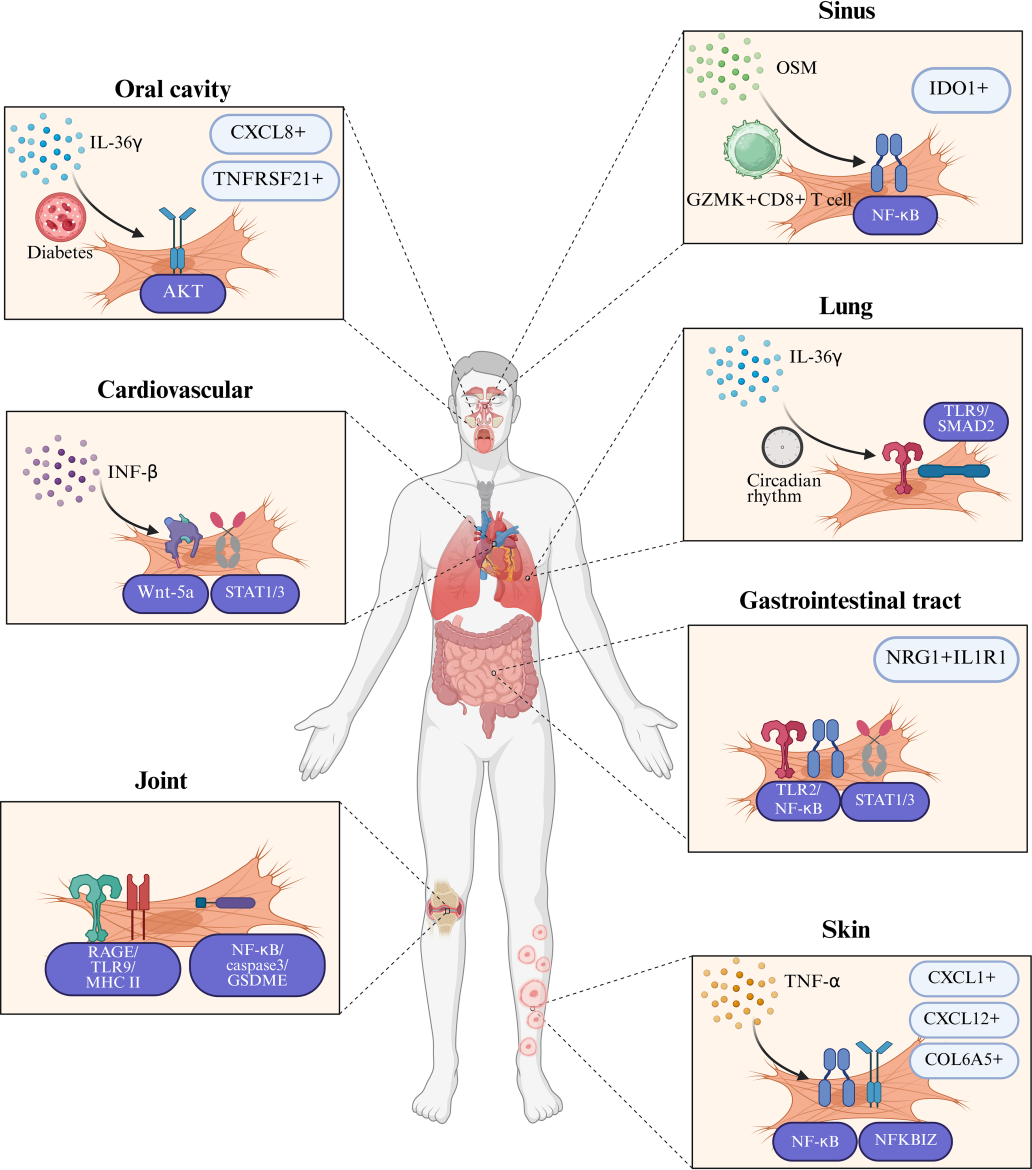
Tissue-specific characteristics in fibroblast-neutrophil communication in inflammatory diseases. This figure illustrates the distinct tissue-specific mechanisms through which fibroblasts and neutrophils interact in the pathogenesis of inflammatory diseases. In chronic obstructive pulmonary disease (COPD), IL-36γ primarily stimulates lung fibroblasts to release IL-1, CXCL1, GM-CSF, and matrix metalloproteinases, thereby promoting both inflammation and fibrosis. In systemic lupus erythematosus (SLE) skin lesions and pulmonary fibrosis, IL-17-containing neutrophil extracellular traps (NETs) activate fibroblasts to produce collagen, contributing to tissue remodeling and fibrosis. Organ-specific fibroblast subpopulations exhibit unique patterns in their interaction with neutrophils: IDO1+ fibroblasts in chronic rhinosinusitis with nasal polyps (CRSwNP) promote neutrophil recruitment by secreting CXCL1/2/3/5/6/8, while TNFRSF21+ fibroblasts in periodontitis facilitate neutrophil infiltration through a distinct chemokine profile. In rheumatoid arthritis (RA), fibroblast-like synoviocytes (FLS) present NET-derived citrullinated peptides via MHC-II, bridging innate and adaptive immunity and promoting chronic inflammation. In inflammatory bowel disease (IBD), NRG1+IL1R1+ fibroblasts are impaired in their reparative functions when exposed to IL-1β released by neutrophils, which exacerbates intestinal inflammation. These tissue-specific interactions are further influenced by systemic factors such as diabetes, which enhances the production of CXCL1/12 through insulin signaling pathways in gingival fibroblasts, and environmental factors such as circadian rhythm disruption, which amplifies CXCL5-mediated neutrophil recruitment in lung fibroblasts through hyperactivation of the NF-κB pathway. Created in BioRender. cai, c. (2025) https://BioRender.com/dovm1ct.

Our systematic analysis of the interactions between fibroblasts and neutrophils across inflammatory diseases in multiple organ systems reveals both common patterns (**Figure 1**) and specific differences in their cellular interactions (**Figure 2**). Inflammatory mediators (IL-17, IL-1β) stimulate fibroblasts to secrete neutrophil-attracting chemokines (CXCL1, CXCL2, CXCL8, CXCL12), which bind to CXCR2 and CXCR4 receptors to drive neutrophil recruitment to inflamed tissues. In turn, neutrophil-derived factors (NETs, elastase, cytokines) modulate fibroblast activity, creating a positive feedback loop that amplifies inflammation. In inflammatory diseases affecting various tissues and organs, these cellular interactions exhibit differences in both mechanism and function. **Table 1** offers a detailed overview of key fibroblast subpopulations identified in inflammatory diseases, including their molecular signatures, functional properties, and interactions with neutrophils. It also includes information on how these fibroblast subsets influence neutrophil recruitment, activation, and function in specific disease contexts.

**Table 1:** Fibroblast subpopulations, their phenotypic characteristics, signaling pathways, and roles in inflammatory diseases, with detailed information on their impact on neutrophil recruitment and function.

Fibroblast Subpopulation	Disease Context	Molecular Signature	Signaling Pathways	Functional Properties	Impact on Neutrophils	Reference
CXCL1+ fibroblasts	Psoriasis, SLE	CXCL1↑, SAA1↑	IL-1β → IL1R1 →NF-κB	Inflammation amplification, recruitment of immune cells	Neutrophil recruitment, activation, delayed apoptosis	(7)
CXCL12+ fibroblasts	Psoriasis, Skin infection	CXCL12↑, NFKBIZ↑, Adipocyte markers↑	IL-17A →IL-17R → NFKBIZ; TNF-α → TNFR1 → NF-κB	Chemokine production, response to IL-17	Neutrophil recruitment, essential for infection clearance	(4)
COL6A5+ fibroblasts	Psoriasis	COL6A5↑, differentiation potential	/	Precursors to CXCL1+ fibroblasts	Indirect effect through differentiation	(7)
Lung fibroblasts	COPD, Pulmonary fibrosis	IL-36R↑, CXCL1/8↑, IL-6↑, MMP2/9↑	IL-36γ → IL-36R/IL-1RAcP complex → NF-κB/MAPK; IL-1β → IL1R1 →NF-κB	Response to IL-36γ, inflammation amplification	Neutrophil recruitment via CXCL1/8	(17, 18)
NRG1+IL1R1+ fibroblasts	Inflammatory bowel disease	NRG1↑, IL1R1↑	IL-1β → IL1R1 → NF-κB; IL-1β → IL1R1 →STAT1/3	Epithelial repair, responds to IL-1β	Impaired reparative function with neutrophil-derived IL-1β	(29)
Fibroblast-like synoviocytes	Rheumatoid arthritis	ACLY↑, Ac-p65↑, inflammatory mediators↑	NETs → ACLY → Ac-p65 → NF-κB	Enhanced proliferation, migration, and invasiveness; metabolic reprogramming	Correlation with NETs in synovial fluid and tissue; linked to disease activity	(39)
Fibroblast-like synoviocytes	Rheumatoid arthritis	MHC-II↑, IL-6↑, IL-8↑, IL-33↑	NETs → RAGE→TLR9 → MHC II	Antigen presentation, inflammatory cytokine production	Neutrophil survival extension, NET induction	(35)
IDO1+ fibroblasts	CRSwNP	IDO1↑, CXCL1/2/3/5/6/8↑	IL-1β → IL-1R → NF-κB	Response to IL-1 signaling, tryptophan metabolism	Neutrophil recruitment	(41)
TNFRSF21+ fibroblasts	Periodontitis	TNFRSF21↑, CXCL1/2/5/6/13↑, IL-24↑	/	Inflammation amplification,B and T cell positioning	Neutrophil recruitment	(49)
CXCL8+ gingival fibroblasts	Peri-implantitis	CXCL8↑	/	Inflammation amplification	Interaction with CXCR2+ neutrophils	(57)
Cardiac fibroblasts	Myocardial infarction, ischemia	MCP-1↑, IP-10↑, TGF-β1↑	IFN-β → IFNAR → JAK → STAT1/3; NETs → Wnt5a	Response to IFN-β, ECM production	Neutrophil migration, resolution of inflammation	(32)

Based on the pathways and molecular mechanisms described previously regarding the involvement of these two cell types in various disease pathological processes, researchers have developed a series of clinical strategies. For instance, **Table 2** lists several anti-fibrotic drugs that have been approved for clinical use or are under clinical trial. Combined targeting of TGF-β signaling and EP300 inhibition (e.g., via metformin) dynamically modulates fibroblast plasticity by suppressing pro-fibrotic myofibroblast differentiation, reversing fibrosis through lipofibroblast induction, and attenuating TGF-β blockade-driven inflammation. This dual strategy disrupts the fibrosis-inflammation cycle, demonstrating cross-organ therapeutic efficacy in pulmonary, cardiac, and vascular fibrosis models, while synergistically enhancing antifibrotic therapies such as pirfenidone in validated systems like WI-38 cells (58).

**Table 2:** Anti-fibrotic drugs targeting fibroblasts: approved for clinical use or under clinical trial, their mechanisms of action, and therapeutic indications.

Drug Name	Mechanism of Action	Indications	Status	Reference
Pirfenidone	Inhibits TGF-β signaling and collagen synthesis.	Idiopathic pulmonary fibrosis, liver fibrosis.	Approved	(79)
Nintedanib	Inhibits multiple kinases involved in fibrosis, including TGF-β.	Idiopathic pulmonary fibrosis, systemic sclerosis.	Approved	(80)
TRK250	Inhibits TGF-β1 expression using siRNA technology, targeting collagen production and fibrosis.	Idiopathic pulmonary fibrosis	Phase I trial completed	(81)
PBI-4050	Inhibits the differentiation of fibroblasts to myofibroblasts, and reduces accumulation of ECM protein deposition and fibrosis.	Idiopathic pulmonary fibrosis	Phase II trial completed, no significant benefit	(82)
BI-1015550	Inhibit TGF-β1induced myofibroblast transformation and ECM deposition	Idiopathic pulmonary fibrosis	Phase III trial ongoing	(83)

Additionally, NETs inhibitors and IL-17 pathway inhibitors have been used in the clinical treatment of inflammatory diseases. Saffarzadeh et al. (59) underscore the importance of developing targeted therapeutic strategies based on the distinct NETosis pathways, such as the ROS-dependent classical pathway and the receptor-triggered rapid pathway. Inhibiting spontaneous NETosis may alleviate tissue damage, while enhancing receptor-mediated NET formation could aid in combating resistant infections. Furthermore, the study clarifies that phagocytosis and NETosis operate synergistically but independently, with no causal dependence between the two processes. Therapeutic strategies targeting NETs focus on suppressing their formation through PAD4 inhibitors such as GSK484, which block histone citrullination, and enhancing their degradation using DNase I to cleave extracellular DNA scaffolds (60). However, systemic toxicity from PAD4 inhibitors and poor tissue penetration of DNase I hinder clinical translation. Clinical evaluation of NETs inhibitors remains limited to a single phase I trial of CIT-013, a humanized monoclonal antibody targeting NETs clearance. This randomized, double-blind, placebo-controlled study in healthy volunteers assessed the agent’s safety, tolerability, and pharmacokinetic profile (61). Results demonstrated that CIT-013 effectively suppressed LPS-induced systemic NET component levels, suggesting its potential therapeutic application in NETs-associated diseases.

In terms of IL-17 pathway inhibitors, a randomized, double-blind pilot study compared the safety and efficacy of two commercialized IL-17 inhibitors, ixekizumab and secukinumab, in the treatment of moderate-to-severe psoriasis (62). The results showed that both groups exhibited a robust clinical response, a significant improvement in patient quality of life, and a satisfactory safety profile, while the ixekizumab group achieved slightly superior outcomes compared to the secukinumab group. Another network meta-analysis compared the time to achieve clinically significant improvement among various biologic agents in patients with moderate-to-severe plaque psoriasis (63). The results indicated that the IL-17 inhibitors brodalumab and bimekizumab demonstrated the fastest onset of action. However, reports indicate that anti-IL-17 therapies targeting specific tissue disorders have exhibited notable adverse effects in other tissues. Real-world evidence demonstrates that 21 cases of IBD emerged in patients undergoing IL-17 therapy with secukinumab and ixekizumab for the treatment of psoriasis, ankylosing spondylitis, and psoriatic arthritis (64). These patients required alternative treatments such as anti-TNF agents. In the cases involving secukinumab, all patients discontinued the medication, and their gastrointestinal symptoms significantly improved (65–74).The cause of these adverse effects is thought to be the reduction in neutrophil recruitment due to anti-IL-17 therapy, which impairs the immune protective function of the intestinal tract (64).This reflects the core findings of this review: while certain mechanistic commonalities exist across inflammatory diseases of various organs in terms of fibroblast recognition of upstream signals such as IL-17 and neutrophil recruitment, heterogeneity exists in aspects such as neutrophil function across different organ tissues.

## 10 Conclusions

This review utilizes evidence from multiple organ disease models to elucidate the crucial role of fibroblast-neutrophil interactions in inflammatory disease development, offering new perspectives for understanding disease pathogenesis. Through systematic comparison, it reveals both common patterns and specific differences in these cellular interactions, providing important insights for future precise interventions in inflammatory diseases.

Despite our comprehensive approach, this review has several limitations. Our analysis is constrained by the available literature, which lacks standardized terminology across different organ systems. We were unable to perform quantitative meta-analysis due to methodological heterogeneity in existing studies.

While our synthesis addresses significant gaps in understanding tissue-specific interactions, important challenges remain in the broader field. The predominant reliance on murine models introduces substantial translational barriers, as these models exhibit marked differences from humans in immune microenvironment characteristics, including neutrophil lifespan, NET formation efficiency, and fibroblast heterogeneity (75–77). Notably, specific chemokine-receptor axes (e.g., CXCL1-CXCR2) play divergent roles in fibroblast-neutrophil interactions across species (78), potentially compromising the cross-species applicability of findings. Furthermore, existing *in vitro* experiments largely employ static co-culture systems or single time-point analyses that fail to capture the dynamic evolution from inflammation to repair and fibrosis.

From a clinical perspective, current therapeutic approaches face challenges of tissue-specific heterogeneity, as evidenced by the paradoxical effects of IL-17 inhibitors that benefit psoriasis but potentially exacerbate intestinal inflammation (64). Additionally, the development of truly precision-targeted therapies is hindered by limited clinical data on NETs inhibitors, inadequate biomarkers for patient stratification, and incomplete understanding of temporal dynamics in disease progression.

Overcoming these challenges requires developing advanced research platforms such as organ-specific 3D co-culture models, implementing spatial-temporal multi-omics analyses, and designing precision-targeted interventions like nano-carriers targeting specific fibroblast subpopulations. These approaches would enable more selective modulation of pathogenic pathways while preserving beneficial immune functions, ultimately facilitating the development of personalized therapeutic strategies tailored to individual patients’ disease profiles.
